# Supplementary material for: The Influence of the Degree of Forest Management on Methylmercury and the Composition of Microbial Communities in the Sediments of Boreal Drainage Ditches
Source: Microorganisms. 2022 Oct 6;10(10):1981. doi: 10.3390/microorganisms10101981 (PMC9609920; doi:10.3390/microorganisms10101981)
Supplement: Supplementary file 1 [file microorganisms-10-01981-s001.zip › microorganisms-1920212-supplementary.pdf]

Supplementary Information

for: **The influence of the degree of forest management on methylmercury and the composition of microbial communities in the sediments of boreal drainage ditches**

Table S1 Topographical characteristics of sub-catchments of watercourses corresponding to each sampling site.

**Parameters of sub-catchment, unit**	**Sampling site**							
	**Site 1**	**Site 2**	**Site 3**	**Site 4**	**Site 5**	**Site 6**	**Site 7**	**Site 8**
Sub-catchment area, ha	27.8	54.6	11.0	70.6	758.5	256.4	314.1	665.2
Elevation (min-max), m	95.8	95.8	99.1	93.1	93.4	92.9	96.4	114.3
Elevation (max), m	98.7	101.7	99.6	106.8	132.3	99.9	132.3	169.1
Total ditch length, km	0.05	0.58	0.00	3.03	63.65	18.69	24.60	12.34
Ditch density, m ha^{-1}	1.9	10.7	0.0	42.9	83.9	72.9	78.3	18.6
Forest area, ha	25.10	51.37	9.42	65.93	717.51	242.37	304.87	452.60
Share of forest area of total sub-catchment area, %	90.1	94.0	85.5	93.3	94.6	94.5	97.1	68.0
Share of spruce-dominated forest from the total forest area, %	27.7	25.8	0.0	13.1	41.2	44.6	31.8	21.0
Share of pine-dominated forest from the total forest area, %	63.5	69.0	74.1	54.8	39.4	38.9	45.8	41.0
Share of broadleaves-dominated forest from the total forest area, %	8.8	5.2	25.9	32.2	16.7	16.5	16.2	30.8

Area of clearfellings in sub-catchment, ha	0.0	0.0	0.0	0.0	19.0	0.0	18.9	14.5
Share of clearfellings from the total forest area, %	0.0	0.0	0.0	0.0	2.6	0.0	6.2	3.2
Share of drained peatlands from the total forest area, %	12.0	9.6	0.0	19.1	35.8	21.7	39.8	2.4
Share of peatland forests from the total forest area, %	40.2	29.6	51.8	0.0	1.1	0.0	2.5	5.0
Share of wetlands from the total forest area, %	0.0	0.0	16.0	0.0	0.5	0.0	0.7	1.0

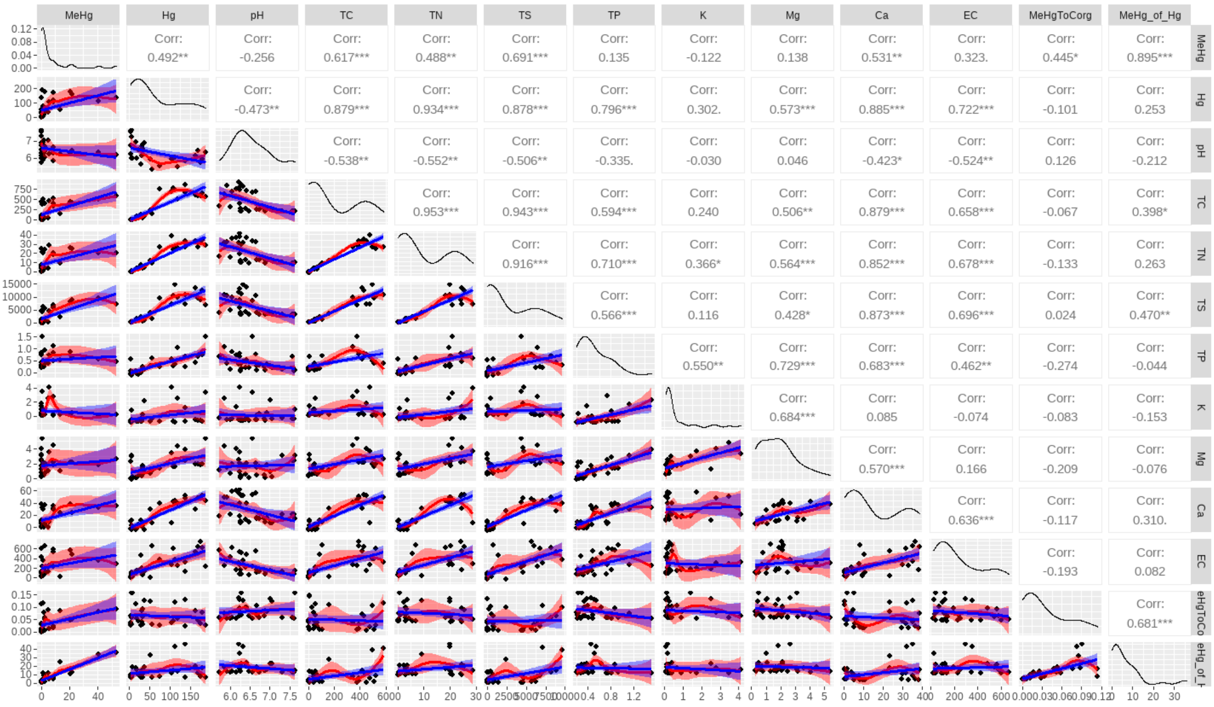

Figure S1 Scatterplot of environmental variables (untransformed) and their correlations. Patterns are visualized with lm and standard error bounds (blue) or loess and a t-based approximation (red).

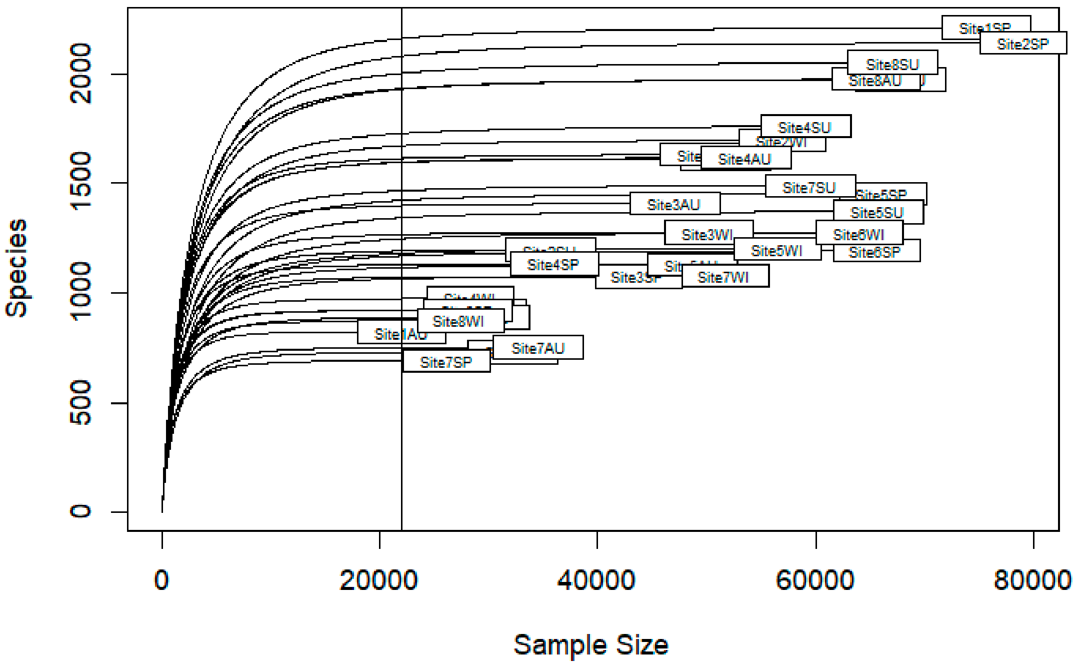

Figure S2 Rarefaction curves and sequencing depth. The vertical abline indicates on the smallest common number of sequencing reads

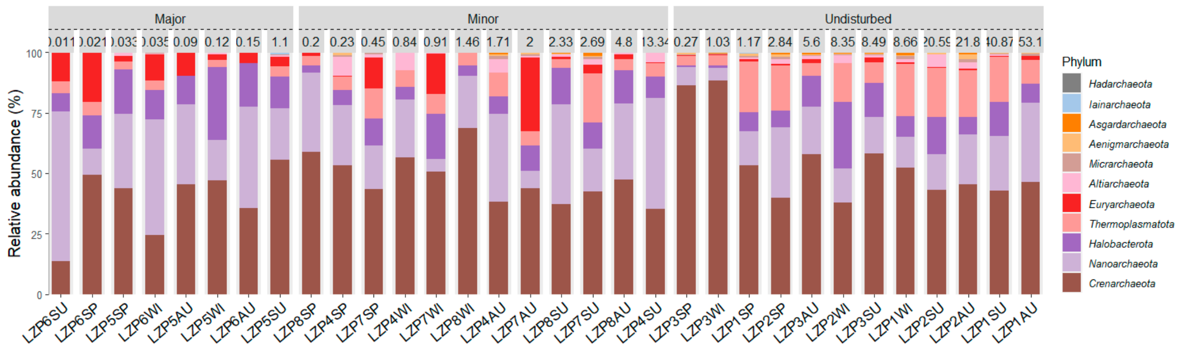

Figure S3 Bar plot of relative abundance of phylum by the level of disturbance. Samples with 15 the most abundant archaeal phyla are grouped by the level of disturbance and sorted from lowest to highest concentration (μg kg^{-1}) of MeHg within each level.

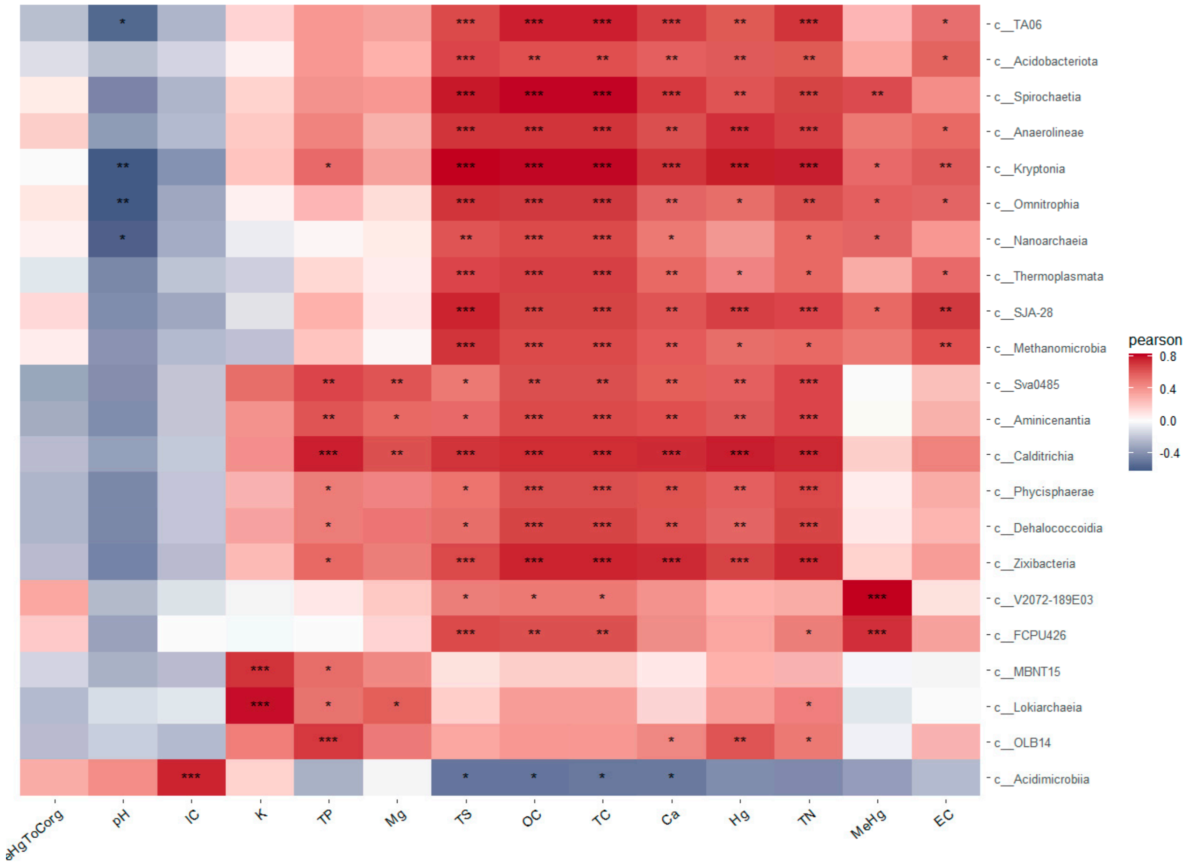

Figure S4 Unconstrained correlations between environmental variables and classes of bacteria and archaea.

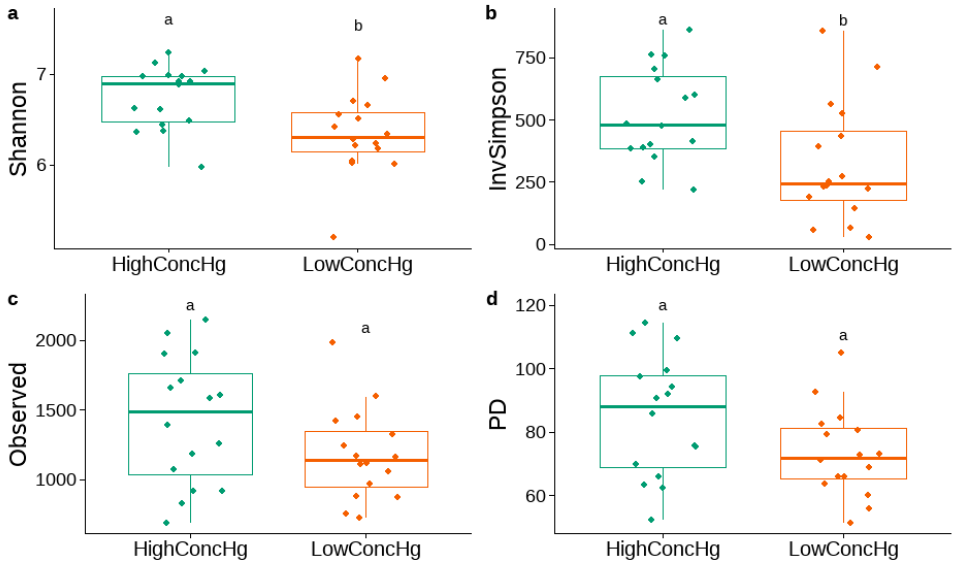

Figure S5 A one-way ANOVA test on high and low concentration of mercury effect on Alpha diversity.

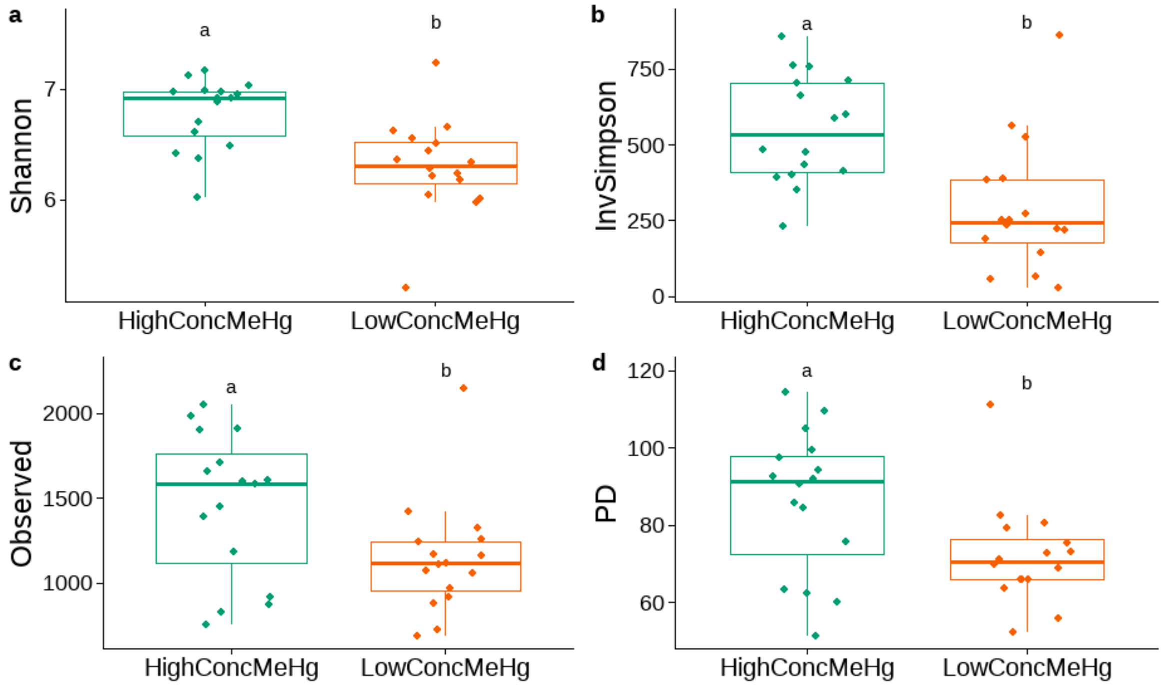

Figure S6 A one-way ANOVA test on high and low concentration of methylmercury effect on Alpha diversity.

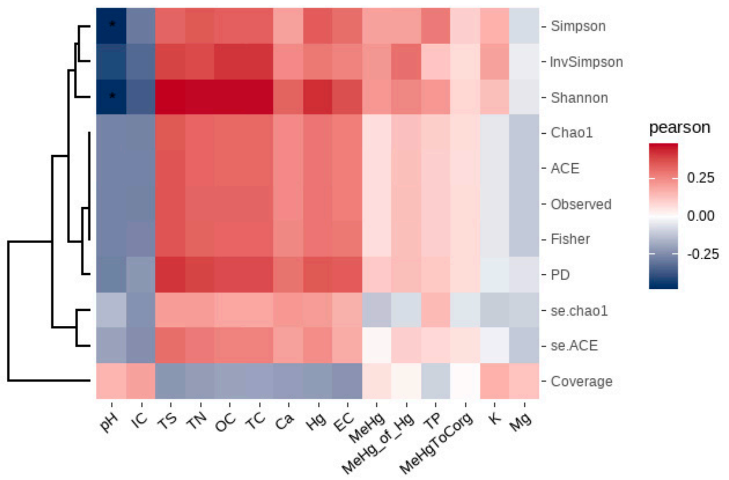

Figure S7 Spearman correlation heatmap between environmental variables and Alpha diversity metrics.

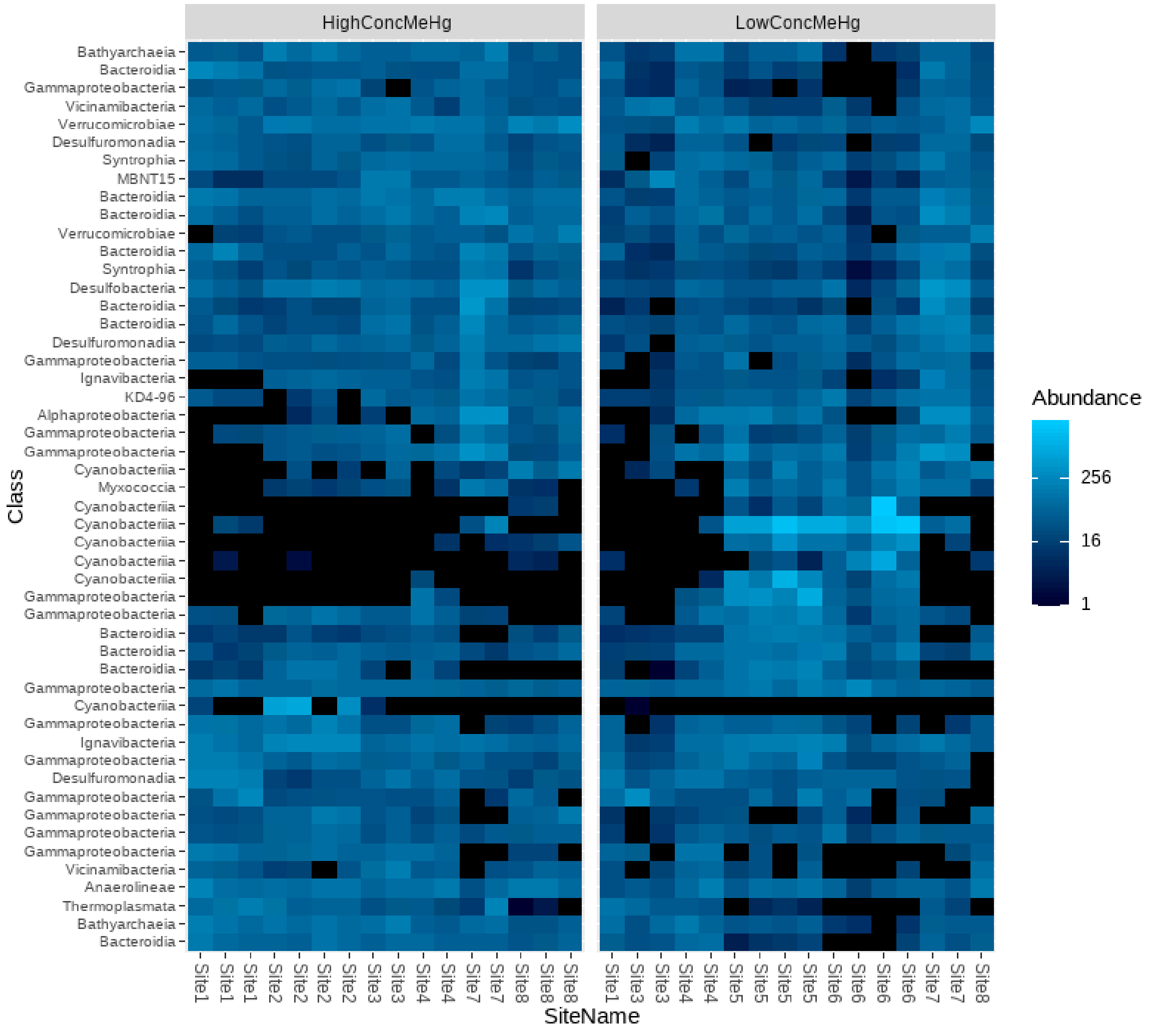

Figure S8 Abundance heatmap of the top 50 most abundant taxa, separated by the level of MeHg. Clustering is based on Bray-Curtis dissimilarity and NMDS ordination.

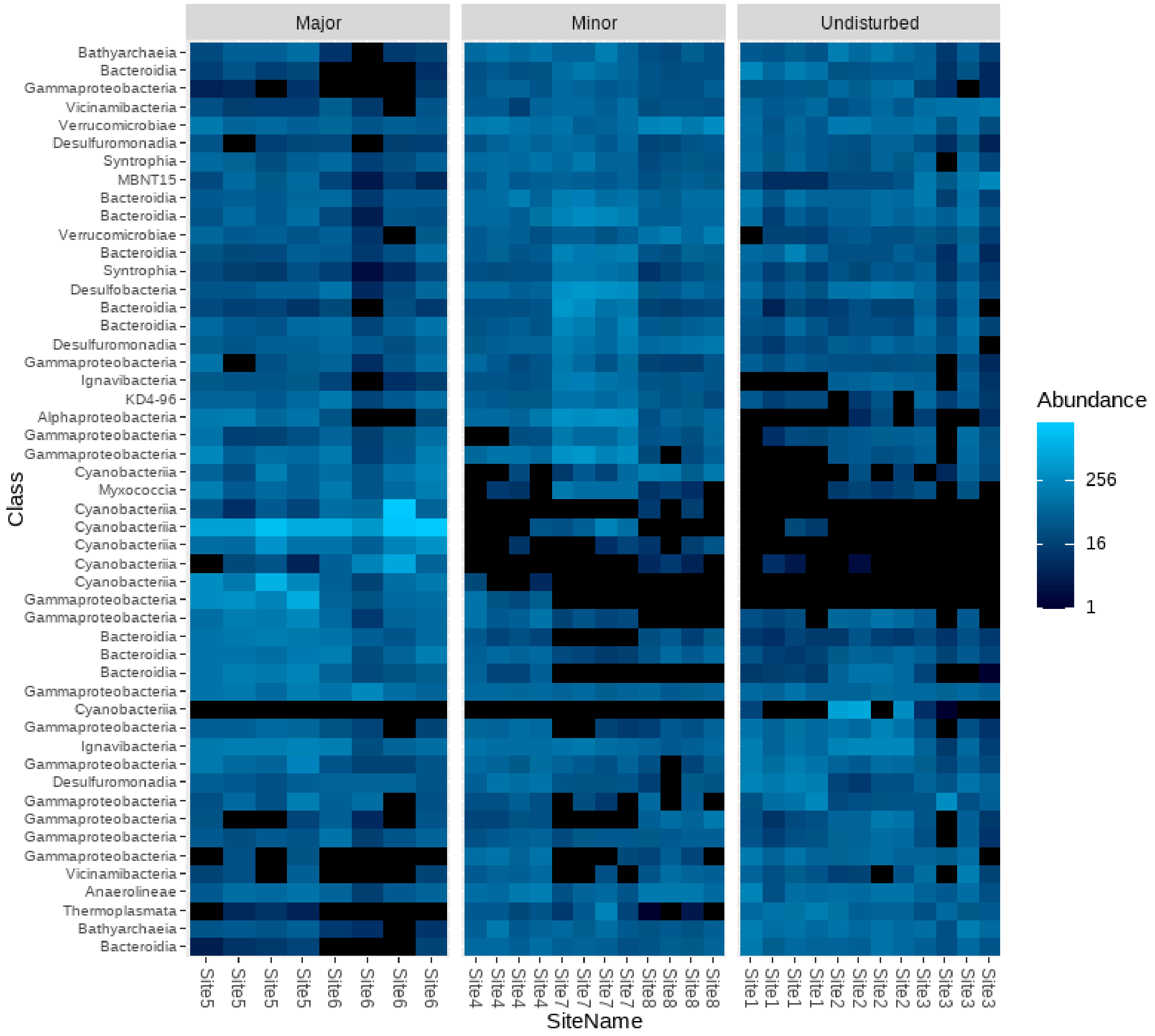

Figure S9 Abundance heatmap of the top 50 most abundant taxa, separated by the level of disturbance. Clustering is based on Bray-Curtis dissimilarity and NMDS ordination.

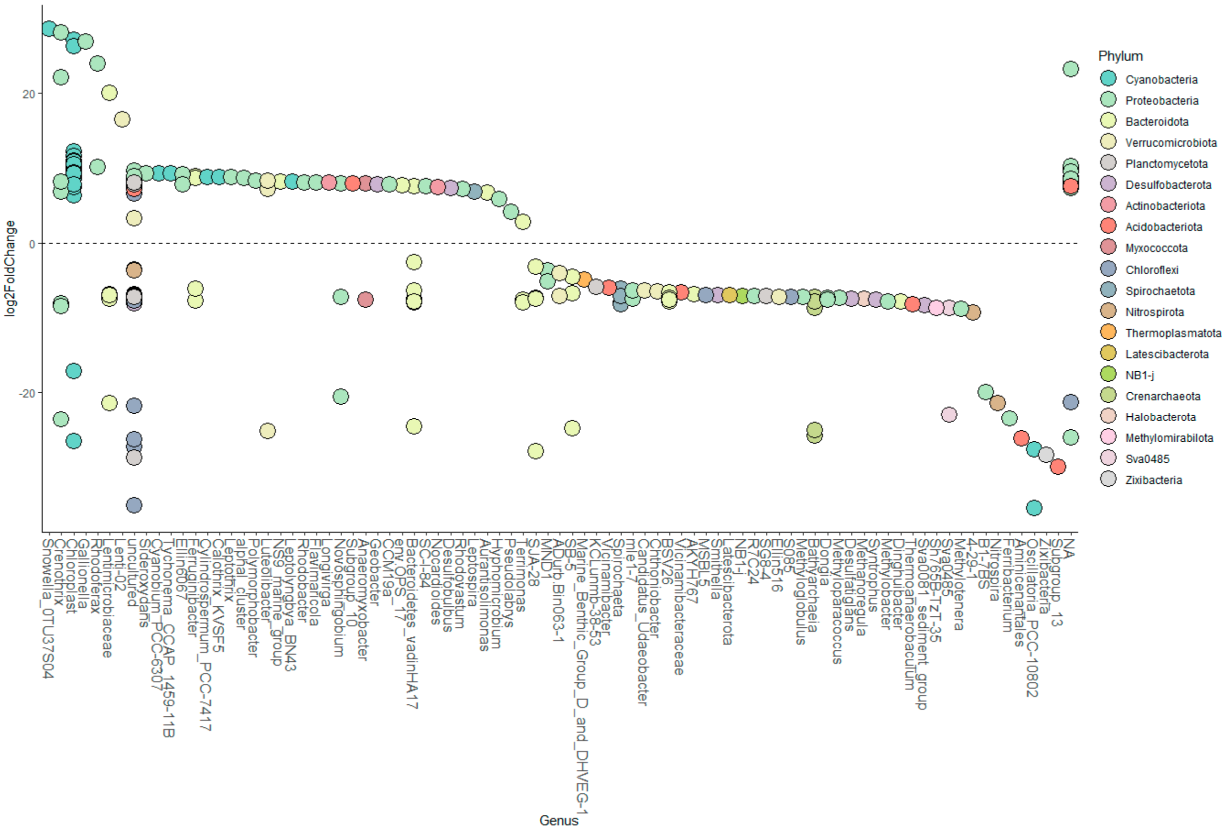

Figure S10 Differential abundance testing using DESeq2. The contrast is set based on contrast between two groups of disturbance levels: Major over Undisturbed. log2FoldChange is the effect size estimate.

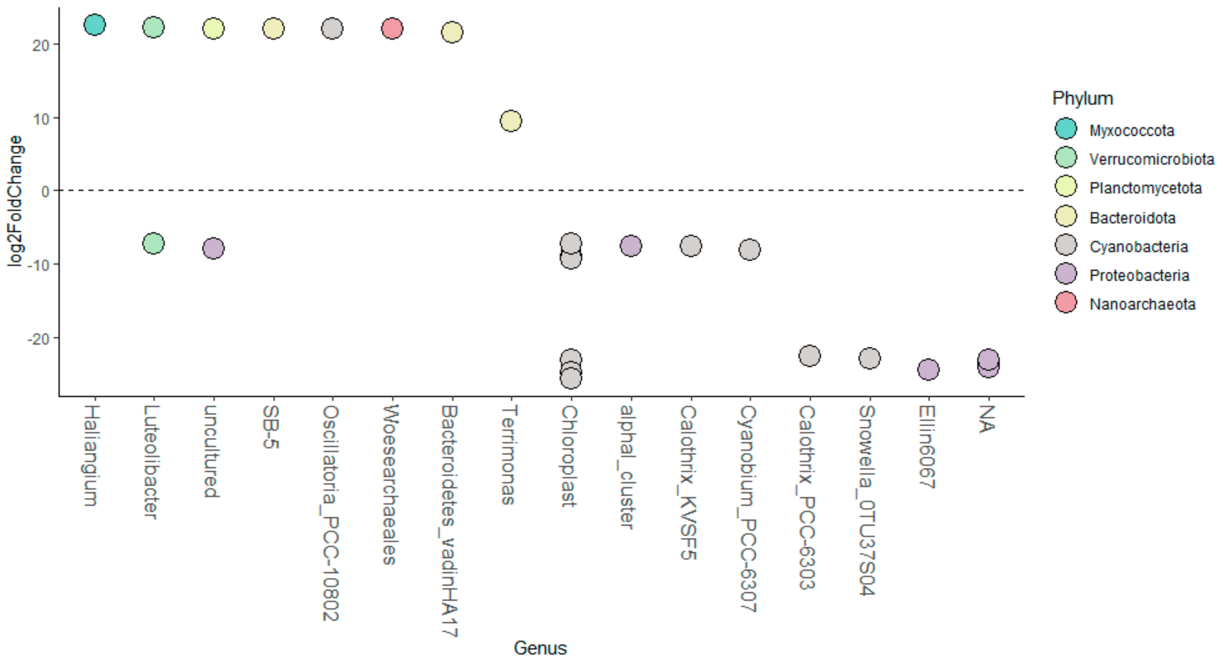

Figure S11 Differential abundance testing using DESeq2. The contrast is set based on median concentration of methylmercury: High concentration over Low concentration. log2FoldChange is the effect size estimate.

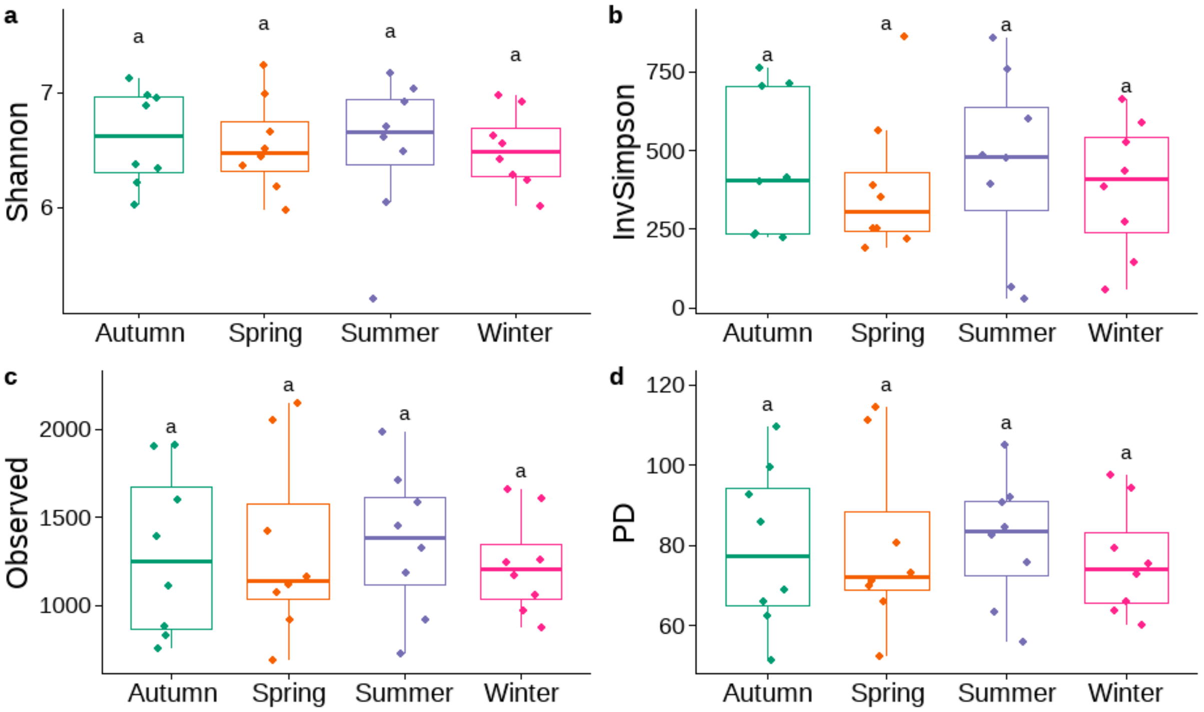

Figure S12 A one-way ANOVA test on seasonal effect on Alpha diversity.

Table S2 Top 10 t indicator value for each level of disturbance

Group:	Minor disturbances								
indval	pval	a.pval	Kingdom	Phylum	Class	Order	Family	Genus	Species
0,88	0,0001	0,0095	d__Bacteria	Acidobacteriota	Holophagae	Holophagales	Holophagaceae	Holophagaceae	metagenome
0,83	0,0001	0,0095	d__Bacteria	Proteobacteria	Gammaproteobacteria	Burkholderiales	Rhodocyclaceae	Dechloromonas	
0,75	0,0001	0,0095	d__Bacteria	Bacteroidota	Bacteroidia	Bacteroidales	SB-5	SB-5	uncultured_bacterium
0,73	0,0001	0,0095	d__Bacteria	Proteobacteria	Gammaproteobacteria	Burkholderiales	Hydrogenophilaceae	Thiobacillus	
0,72	0,0003	0,0190	d__Bacteria	Desulfobacterota	Syntrophorhabdia	Syntrophorhabdales	Syntrophorhabdaceae	Syntrophorhabdus	uncultured_delta
0,71	0,0044	0,0885	d__Bacteria	Bacteroidota	Bacteroidia	Bacteroidetes_VC2.1_Bac22	Bacteroidetes_VC2.1_Bac22	Bacteroidetes_VC2.1_Bac22	uncultured_soil
0,71	0,0032	0,0732	d__Bacteria	Bacteroidota	Bacteroidia	Bacteroidales	Bacteroidetes_vadinHA17	Bacteroidetes_vadinHA17	
0,71	0,0003	0,0190	d__Bacteria	NB1-j	NB1-j	NB1-j	NB1-j	NB1-j	
0,70	0,0090	0,1547	d__Bacteria	Bacteroidota	Bacteroidia	Sphingobacteriales	Lentimicrobiaceae	Lentimicrobiaceae	uncultured_bacterium
0,70	0,0014	0,0464	d__Bacteria	Proteobacteria	Alphaproteobacteria	Rhizobiales	Xanthobacteraceae	Pseudolabrys	
Group:	Major disturbance								
indval	pval	a.pval	Kingdom	Phylum	Class	Order	Family	Genus	Species
1,00	0,0001	0,0095	d__Bacteria	Cyanobacteria	Cyanobacteriia	Synechococcales	Cyanobiaceae	Cyanobium_PCC-6307	
1,00	0,0001	0,0095	d__Bacteria	Cyanobacteria	Cyanobacteriia	Oxyphotobacteria	Unknown_Family	Calothrix_KVSF5	uncultured_bacterium
1,00	0,0001	0,0095	d__Bacteria	Proteobacteria	Gammaproteobacteria	Burkholderiales	Comamonadaceae		
1,00	0,0001	0,0095	d__Bacteria	Proteobacteria	Gammaproteobacteria	Burkholderiales	Nitrosomonadaceae	Ellin6067	
1,00	0,0001	0,0095	d__Bacteria	Cyanobacteria	Cyanobacteriia	Chloroplast	Chloroplast	Chloroplast	Planoglabratella_opercularis
1,00	0,0001	0,0095	d__Bacteria	Proteobacteria	Gammaproteobacteria	Burkholderiales	Comamonadaceae	Rhodoferax	
1,00	0,0001	0,0095	d__Bacteria	Cyanobacteria	Cyanobacteriia	Chloroplast	Chloroplast	Chloroplast	
0,99	0,0001	0,0095	d__Bacteria	Cyanobacteria	Cyanobacteriia	Chloroplast	Chloroplast	Chloroplast	Planoglabratella_opercularis
0,99	0,0001	0,0095	d__Bacteria	Chloroflexi	Anaerolineae	Caldilineales	Caldilineaceae	uncultured	metagenome

0,98	0,0001	0,0095	d__Bacteria	Desulfobacterota	Desulfuromonadia	Geobacterales	Geobacteraceae	Geobacter	
Group:	Undisturbed								
indval	pval	a.pval	Kingdom	Phylum	Class	Order	Family	Genus	Species
1,00	0,0001	0,0095	d__Bacteria	Bacteroidota	Kryptonia	Kryptoniales	BSV26	BSV26	uncultured_bacterium
0,93	0,0001	0,0095	d__Bacteria	Bacteroidota	Bacteroidia	Sphingobacteriales	Lentimicrobiaceae	Lentimicrobiaceae	
0,92	0,0001	0,0095	d__Archaea	Crenarchaeota	Bathyarchaeia	Bathyarchaeia	Bathyarchaeia	Bathyarchaeia	uncultured_methanogenic
0,92	0,0001	0,0095	d__Bacteria	Proteobacteria	Alphaproteobacteria	Dongiales	Dongiaceae	Dongia	metagenome
0,92	0,0001	0,0095	d__Bacteria	Chloroflexi	Dehalococcoidia	MSBL5	MSBL5	MSBL5	uncultured_bacterium
0,89	0,0001	0,0095	d__Bacteria	Bacteroidota	Bacteroidia	Bacteroidales	Bacteroidetes_vadinHA17	Bacteroidetes_vadinHA17	
0,86	0,0001	0,0095	d__Archaea	Crenarchaeota	Bathyarchaeia	Bathyarchaeia	Bathyarchaeia	Bathyarchaeia	uncultured_methanogenic
0,83	0,0001	0,0095	d__Bacteria	Spirochaetota	Spirochaetia	Spirochaetales	Spirochaetaceae	Spirochaeta	
0,83	0,0001	0,0095	d__Bacteria	Desulfobacterota	Desulfobacteria	Desulfatiglandales	Desulfatiglandaceae	Desulfatiglans	
0,83	0,0001	0,0095	d__Bacteria	Chloroflexi	Dehalococcoidia	S085	S085	S085	uncultured_bacterium

Table S3 Top 10 indicator species with highest indicator value for high and low concentration of methylmercury.

Group:	High concentration MeHg								
indval	pval	a.pval	Kingdom	Phylum	Class	Order	Family	Genus	Species
0,81	0,0002	0,4977	d__Bacteria	Bacteroidota	Bacteroidia	Bacteroidales	SB-5	SB-5	uncultured_bacterium
0,78	0,0001	0,4977	d__Bacteria	Bacteroidota	SJA-28	SJA-28	SJA-28	SJA-28	uncultured_Chlorobi
0,77	0,0005	0,4977	d__Bacteria	Bacteroidota	Bacteroidia	Bacteroidales	SB-5	SB-5	uncultured_bacterium
0,74	0,0003	0,4977	d__Bacteria	Nitrospirota	4-29-1	4-29-1	4-29-1	4-29-1	uncultured_Nitrospirae
0,72	0,0001	0,4977	d__Bacteria	Latescibacterota	Latescibacterota	Latescibacterota	Latescibacterota	Latescibacterota	uncultured_soil

0,71	0,0010	0,5318	d__Bacteria	Bacteroidota	Bacteroidia	Bacteroidales	Bacteroidetes_vadinHA17	Bacteroidetes_vadinHA17	uncultured_bacterium
10,71	0,0003	0,4977	d__Bacteria	Bacteroidota	SJA-28	SJA-28	SJA-28	SJA-28	metagenome
0,70	0,0003	0,4977	d__Archaea	Crenarchaeota	Bathyarchaeia	Bathyarchaeia	Bathyarchaeia	Bathyarchaeia	
0,70	0,0007	0,4977	d__Bacteria	Bacteroidota	Bacteroidia	Bacteroidales	Bacteroidetes_vadinHA17	Bacteroidetes_vadinHA17	uncultured_Alkaliflexus
0,69	0,0011	0,5318	d__Bacteria	Verrucomicrobiota	Verrucomicrobiae	Pedosphaerales	Pedosphaeraceae	ADurb.Bin063-1	uncultured_bacterium
Group:	Low concentration MeHg								
indval	pval	a.pval	Kingdom	Phylum	Class	Order	Family	Genus	Species
0,75	0,0022	0,5713	d__Bacteria	Bacteroidota	Bacteroidia	Cytophagales	Spirosomaceae	Lacihabitans	uncultured_bacterium
0,72	0,0074	0,6881	d__Bacteria	Bacteroidota	Bacteroidia	Flavobacteriales	Flavobacteriaceae	Flavobacterium	
0,68	0,0005	0,4977	d__Bacteria	Bacteroidota	Bacteroidia	Sphingobacteriales	AKYH767	AKYH767	uncultured_bacterium
0,67	0,0015	0,5494	d__Bacteria	Cyanobacteria	Cyanobacteriia	Chloroplast	Chloroplast	Chloroplast	
0,65	0,0283	0,9737	d__Bacteria	Bacteroidota	Bacteroidia	Chitinophagales	Saprospiraceae	Haliscomenobacter	
0,65	0,0029	0,5746	d__Bacteria	Bacteroidota	Bacteroidia	Chitinophagales	Saprospiraceae	uncultured	metagenome
0,65	0,0068	0,6881	d__Bacteria	Actinobacteriota	Actinobacteria	PeM15	PeM15	PeM15	metagenome
0,64	0,0307	0,9769	d__Bacteria	Bacteroidota	Bacteroidia	Chitinophagales	Saprospiraceae	uncultured	
0,63	0,0010	0,5318	d__Bacteria	Chloroflexi	Anaerolineae	Caldilineales	Caldilineaceae	uncultured	metagenome
0,63	0,0004	0,4977	d__Bacteria	Proteobacteria	Alphaproteobacteria	Rhizobiales	Rhizobiales_Incertae_Sedis	uncultured	metagenome

Table S4 Top 10 indicator species with highest indicator value for high and low concentration of mercury.

Group:	High concentration Hg								
indval	pval	a.pval	Kingdom	Phylum	Class	Order	Family	Genus	Species
0,76	0,0002	0,1450	d__Bacteria	Bacteroidota	Bacteroidia	Bacteroidales	Bacteroidetes_vadinHA17	Bacteroidetes_vadinHA17	
0,75	0,0002	0,1450	d__Bacteria	Nitrospirota	4-29-1	4-29-1	4-29-1	4-29-1	uncultured_Nitrospirae
0,75	0,0001	0,1209	d__Bacteria	Bacteroidota	Kryptonia	Kryptoniales	BSV26	BSV26	uncultured_bacterium
0,73	0,0018	0,3326	d__Bacteria	Bacteroidota	SJA-28	SJA-28	SJA-28	SJA-28	uncultured_Chlorobi
0,72	0,0004	0,1667	d__Bacteria	Proteobacteria	Gammaproteobacteria	Burkholderiales	Rhodocyclaceae	Sterolibacterium	uncultured_bacterium
0,72	0,0012	0,3081	d__Archaea	Crenarchaeota	Bathyarchaeia	Bathyarchaeia	Bathyarchaeia	Bathyarchaeia	uncultured_methanogenic
0,72	0,0002	0,1450	d__Bacteria	Myxococcota	Myxococcia	Myxococcales	Anaeromyxobacteraceae	Anaeromyxobacter	uncultured_delta
0,71	0,0012	0,3081	d__Bacteria	Bacteroidota	Bacteroidia	Flavobacteriales	Flavobacteriaceae	Flavobacterium	
0,71	0,0003	0,1450	d__Bacteria	Spirochaetota	Spirochaetia	Spirochaetales	Spirochaetaceae	Spirochaeta	uncultured_bacterium
0,71	0,0009	0,2530	d__Bacteria	Spirochaetota	Spirochaetia	Spirochaetales	Spirochaetaceae	Spirochaeta	Spirochaeta_sp.
Group:	Low concentration Hg								
indval	pval	a.pval	Kingdom	Phylum	Class	Order	Family	Genus	Species
0,89	0,0001	0,1209	d__Bacteria	Proteobacteria	Alphaproteobacteria	Rhodobacterales	Rhodobacteraceae	Flavimaricola	uncultured_bacterium
0,78	0,0001	0,1209	d__Bacteria	Verrucomicrobiota	Verrucomicrobiae	Verrucomicrobiales	Rubritaleaceae	Luteolibacter	
0,78	0,0005	0,1777	d__Bacteria	Verrucomicrobiota	Verrucomicrobiae	Verrucomicrobiales	Verrucomicrobiaceae	uncultured	
0,77	0,0001	0,1209	d__Bacteria	Bacteroidota	Bacteroidia	Chitinophagales	Chitinophagaceae	Aurantisolimonas	
0,75	0,0002	0,1450	d__Bacteria	Planctomycetota	Planctomycetes	Gemmatales	Gemmataceae	uncultured	metagenome
0,75	0,0007	0,2014	d__Bacteria	Verrucomicrobiota	Verrucomicrobiae	Verrucomicrobiales	Verrucomicrobiaceae	uncultured	uncultured_soil

0,74	0,0006	0,1813	d__Bacteria	Cyanobacteria	Cyanobacteriia	Chloroplast	Chloroplast	Chloroplast	
0,74	0,0001	0,1209	d__Bacteria	Cyanobacteria	Cyanobacteriia	Chloroplast	Chloroplast	Chloroplast	
0,71	0,0287	0,7492	d__Bacteria	Desulfobacterota	Desulfobacteria	Desulfobacterales	Desulfosarcinaceae	Desulfatirhabdium	uncultured_bacterium
0,71	0,0054	0,4144	d__Bacteria	Proteobacteria	Gammaproteobacteria	Methylococcales	Methylomonadaceae	Methylobacter	uncultured_bacterium

Table S5. Summary of indicator species affiliation to phyla. The table shows a number of indicator species in each phyla. Total number of indicator species is 223, 161 and 674 for mercury, methylmercury and the level of disturbance, respectively.

	Phylum	High conc. Hg	Low conc. Hg
1	Proteobacteria	30	32
2	Bacteroidota	29	20
3	Cyanobacteria	0	17
4	Chloroflexi	7	7
5	Verrucomicrobiota	6	8
6	Desulfobacterota	8	5
7	Acidobacteriota	8	3
8	Actinobacteriota	2	7
9	Crenarchaeota	6	1
10	Myxococcota	2	5
11	Spirochaetota	6	0
12	Planctomycetota	1	4
13	Nitrospirota	2	0
14	Latescibacterota	2	0
15	Thermoplasmatota	1	0
16	Firmicutes	0	1
17	Sva0485	1	0
18	Abditibacteriota	0	1
19	Methylomirabilota	1	0
	Phylum	High conc. MeHg	Low conc. MeHg
1	Bacteroidota	42	13
2	Proteobacteria	19	13
3	Chloroflexi	10	2
4	Verrucomicrobiota	7	3
5	Desulfobacterota	9	0
6	Cyanobacteria	0	9
7	Acidobacteriota	5	1
8	Crenarchaeota	4	0
9	Latescibacterota	3	0
10	Planctomycetota	2	1
11	Myxococcota	1	2
12	Halobacterota	3	0
13	Nitrospirota	2	0
14	Thermoplasmatota	2	0
15	Spirochaetota	2	0

16	Actinobacteriota	1	1
17	Fibrobacterota	1	0
18	Gemmatimonadota	1	0
19	NB1-j	1	0
20	Zixibacteria	1	0

	Phylum	Minor	Major	Undisturbed
1	Proteobacteria	32	92	43
2	Bacteroidota	34	67	57
3	Cyanobacteria	0	71	1
4	Verrucomicrobiota	15	26	11
5	Planctomycetota	6	27	6
6	Chloroflexi	3	13	23
7	Acidobacteriota	19	5	9
8	Desulfobacterota	7	4	10
9	Actinobacteriota	4	14	1
10	Crenarchaeota	2	0	13
11	Spirochaetota	0	1	11
12	Myxococcota	0	6	4
13	Latescibacterota	1	1	4
14	Thermoplasmatota	0	0	6
15	Sva0485	1	0	3
16	Methylomirabilota	2	0	1
17	RCP2-54	2	0	0
18	Firmicutes	1	1	0
19	Gemmatimonadota	0	1	1
20	Nitrospirota	0	0	2
21	Halobacterota	0	0	2
22	NB1-j	1	0	0
23	Nitrospinota	1	0	0
24	Dadabacteria	1	0	0
25	Nanoarchaeota	0	1	0
26	Bdellovibrionota	0	0	1
27	Fibrobacterota	0	0	1
28	Iainarchaeota	0	0	1
29	LCP-89	0	0	1
